# Supplementary material for: Van der Waals pressure and its effect on trapped interlayer molecules
Source: Nat Commun. 2016 Jul 7;7:12168. doi: 10.1038/ncomms12168 (PMC4941049; doi:10.1038/ncomms12168)
Supplement: Supplementary Information — Supplementary Figures 1-14, Supplementary Notes 1-10 and Supplementary References. [file ncomms12168-s1.pdf]

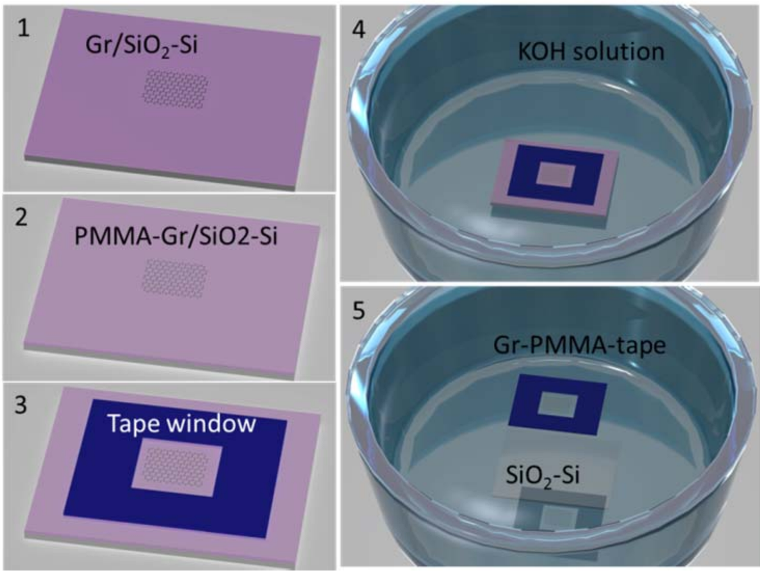

Supplementary Figure 1 | Wet transfer of graphene. Schematic illustration of the steps used for producing a top layer of graphene (Gr) needed for fabrication of an encapsulated sample. Panel numbers indicate the sequence of the steps in the process (1-5).

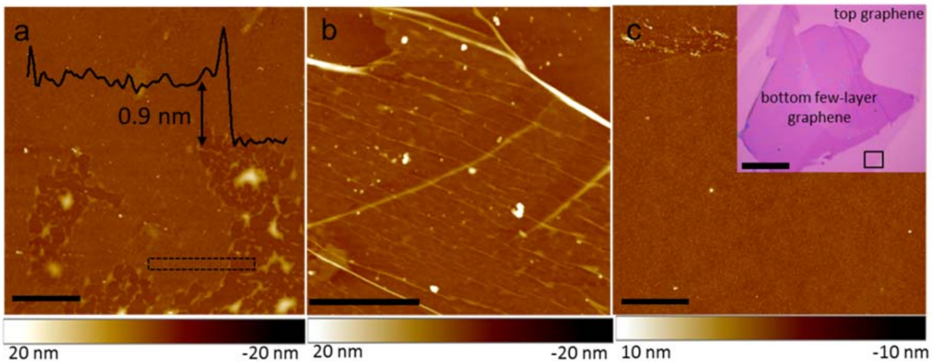

Supplementary Figure 2│AFM images taken for graphene-encapsulated salt and molecule on few-layer graphene. (a) AFM image showing a 0.9-nm-thick, large, flat region in a GE-BA sample. Dark curve: height profile along the dotted rectangle. (b) AFM image showing wrinkle features within the flat 1-nm-thick $MgCl_2$-captured region in a GE-$MgCl_2$ sample. (c) AFM image in the same GE-$MgCl_2$ sample as shown in Supplementary Figure 2b showing absence of bubbles or flat encapsulated regions outside of the graphene-graphene enclosure (in an area where the top single layer of graphene lies on the Si/SiO_2 substrate). All scale bars are 1 μm. Inset shows the optical micrograph of the GE-$MgCl_2$ sample on few-layer graphene (scale bar 30 μm), where the square marked region indicates the area from which the AFM image was obtained.

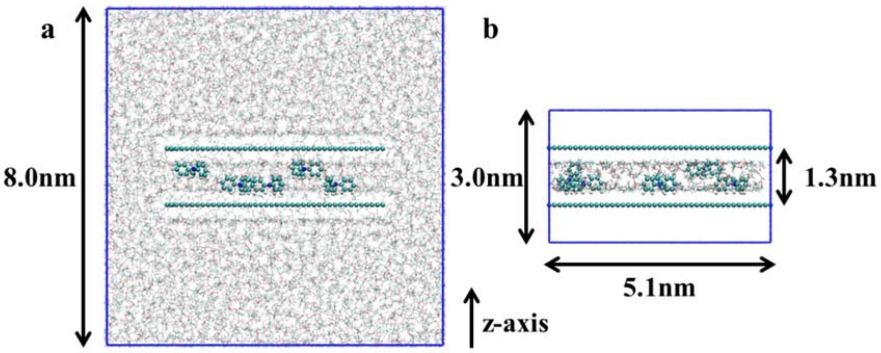

Supplementary Figure 3 | Starting configurations of the molecular dynamics simulation. The images show the starting configurations for the reservoir simulation (a) and the infinite channel simulation (b) for GE-TPA molecules in the presence of methanol. Solid blue lines mark the edge of the simulation cell. The dimensions of the box are 8 nm × 8 nm × 8 nm for **a** and 5.1 nm × 5.1 nm × 3.0 nm for **b**. Separation between graphene sheets in both images is 1.3 nm. The z-axis is defined as perpendicular to the graphene sheets. Color key: C – Cyan, H – White, N – Blue, O – Red.

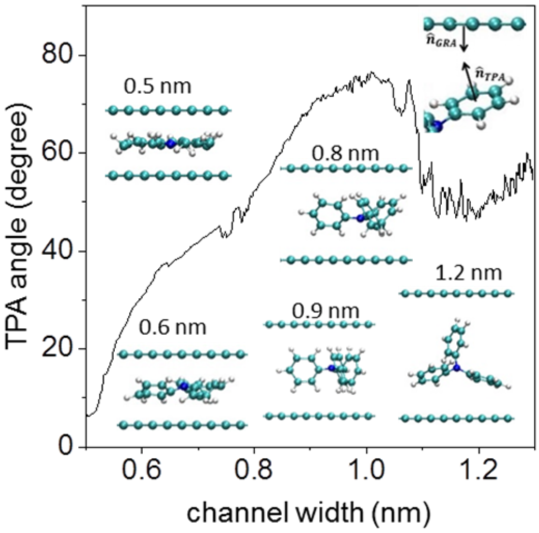

Supplementary Figure 4 │ Confinement-induced effects on TPA measured using MD simulations. Graph shows TPA angle measured as the angle between the vector normal to the TPA phenyl rings and the vector normal to the graphene sheet. Top right inset shows an example of these two vector normals. This TPA angle is average over all phenyl rings for all of the TPA molecules in the simulation. All other insets show the structure of TPA inside graphene capillary under different confinement separations (0.5 – 1.2 nm).

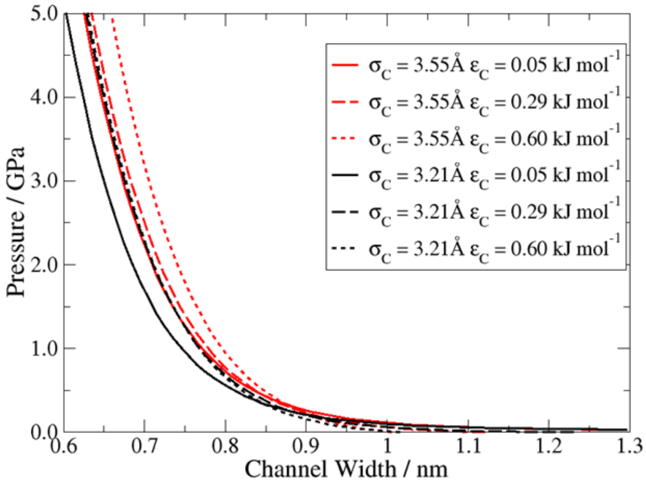

Supplementary Figure 5 | Effects of graphene-molecule interaction on pressure. Graph shows the pressure measured in simulation against the channel width for different graphene interaction parameters.

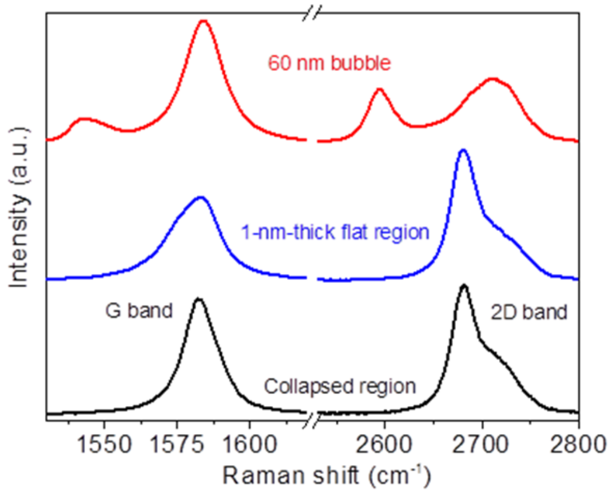

Supplementary Figure 6 | Strain induced shift in the Raman spectra of graphene. Raman spectrum (514 nm excitation) of graphene (collectively from top single layer and bottom few-layers) obtained from a collapsed region, 1-nm-thick flat enclosed region and nanobubble of height 60 nm in a GE-BA sample showing the strain induced shift in the top layer graphene.

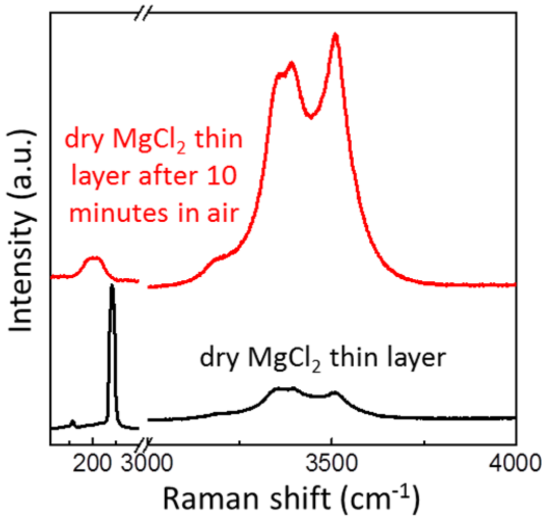

Supplementary Figure 7 | Raman spectrum of hydrated $MgCl_2$. Raman spectrum (488 nm excitation) obtained from a thin layer of $MgCl_2$ deposited on a Si/SiO_2 substrate by drop casting 0.1 M $MgCl_2$ solution immediately after vacuum drying and after 10 min of exposure to air.

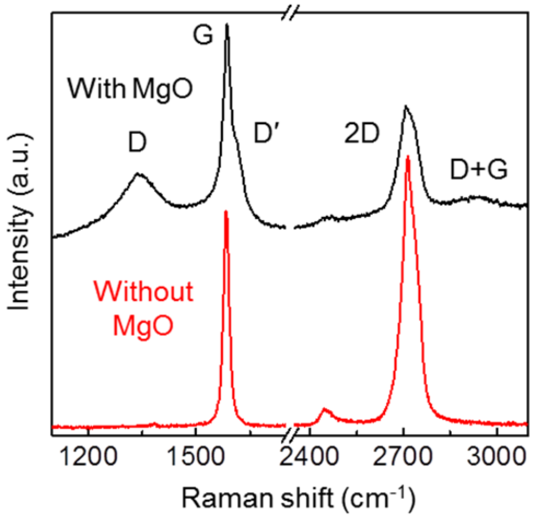

Supplementary Figure 8 | Defect formation in graphene. Raman spectrum (488 nm excitation) of graphene (from both the top single layer and bottom few layers) obtained from GE-$MgCl_2$ sample in the presence and absence of MgO, showing the formation of defects in the graphene when MgO is formed in the enclosure.

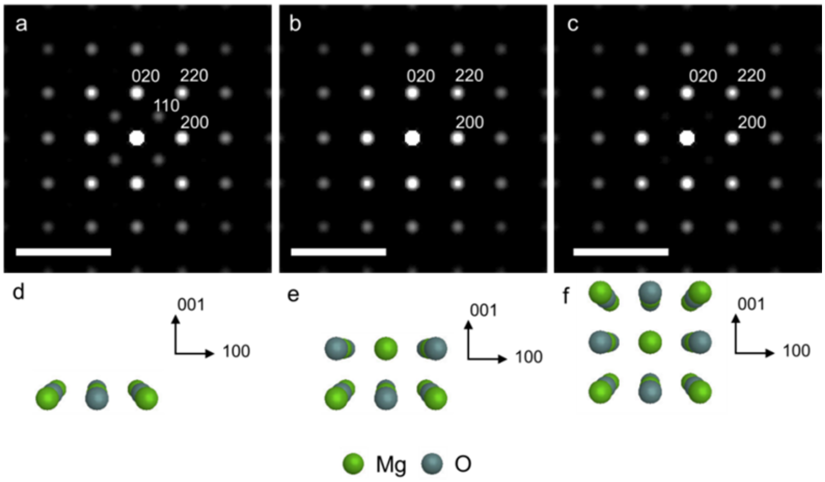

Supplementary Figure 9 | Simulations of the MgO electron diffraction pattern: (a) Simulated electron diffraction patterns from single-layer MgO (b) bilayer MgO and (c) trilayer MgO oriented along the [001] zone axis (scale bar 10 nm^{-1}). (d, e and f) Atomic models used for simulating single layer, bilayer and trilayer MgO, respectively.

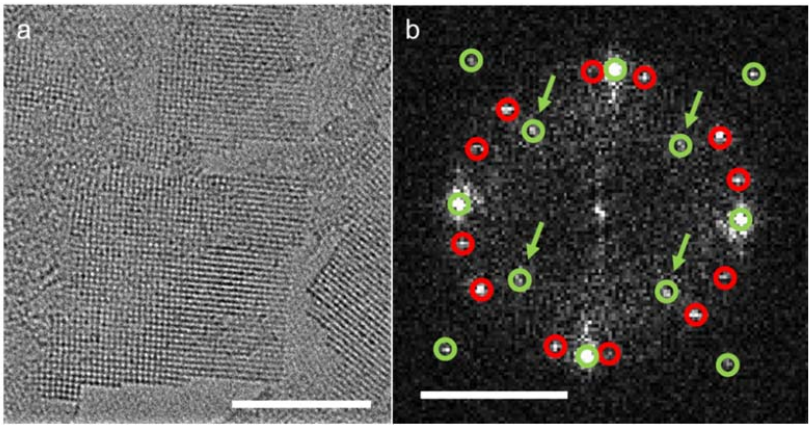

Supplementary Figure 10 | HRTEM imaging of the thinnest MgO crystallites: (a) HRTEM image of the thinnest MgO crystallites found encapsulated between two graphene sheets (scale bar 5 nm). (b) Fast Fourier transform (FFT) obtained from the image in Supplementary Figure 10a showing lattice reflections from the two graphene sheets (red circles) and from the MgO crystals (green circles). The arrows indicate the presence of a (110) lattice reflection which is usually forbidden for bulk MgO (scale bar 5 nm^{-1}).

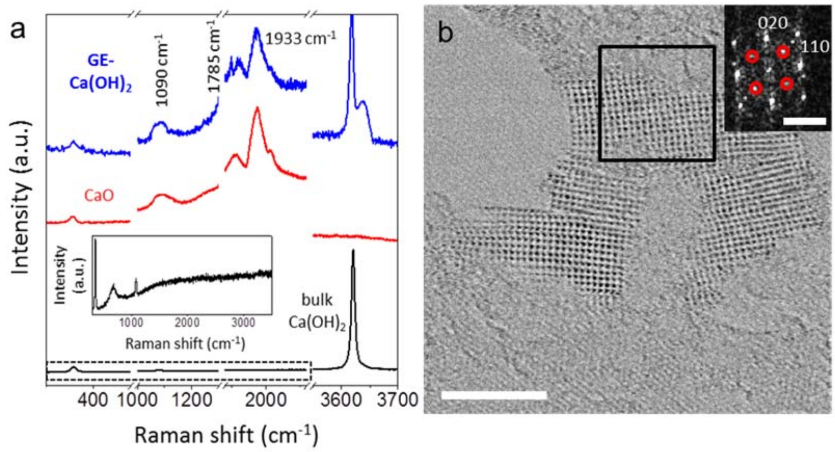

Supplementary Figure 11 | Graphene-encapsulation-induced conversion of $Ca(OH)_2$ to CaO: (a) Raman spectrum (633 nm excitation) of bulk $Ca(OH)_2$, reference CaO synthesized by annealing $Ca(OH)_2$ in vacuum at 350 ^{o}C and GE-$Ca(OH)_2$. The peak positions of all observed PL bands from CaO are marked. Inset shows the zoomed Raman spectrum of bulk $Ca(OH)_2$ obtained from the marked region. (b) HRSTEM BF image showing the square lattice of CaO in the GE-$Ca(OH)_2$ sample (scale bar 2 nm). Inset shows the FFT from the marked region in the main figure, red circles indicate (110) lattice reflections of CaO (scale bar 5 nm^{-1}).

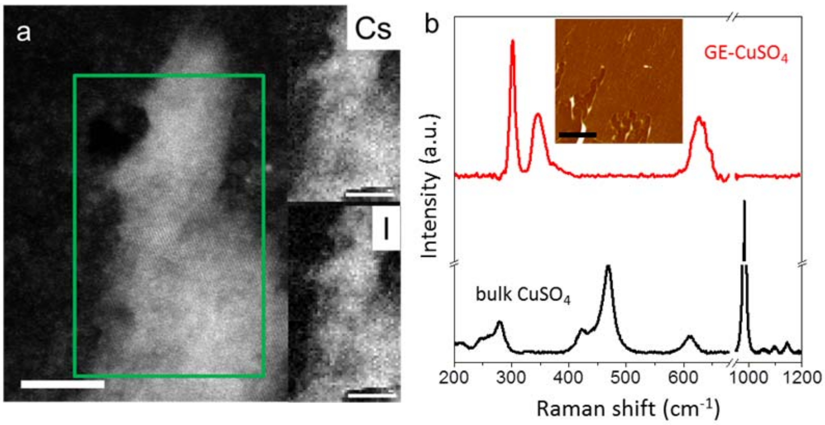

Supplementary Figure 12│Graphene-encapsulation effects on CsI and $CuSO_4$ samples: (a) HAADF STEM image showing a GE-CsI crystal. Inset shows EELS mapping of Cs and I, confirming the co-location of these two elements in the sample (all scale bars are 10 nm). Mapped area is indicated by the green rectangle in the main figure. (b) Raman spectrum (514 nm excitation) of bulk $CuSO_4$ and GE-$CuSO_4$. Inset shows the AFM image of 1-nm-thick, flat, encapsulated regions of $CuSO_4$ (scale bar 1 μm).

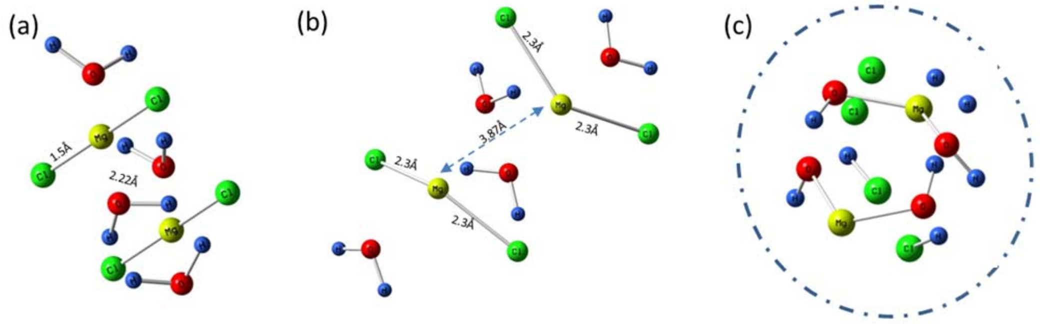

Supplementary Figure 13 | $MgCl_2$ under confinement. (a) Initial configuration of compact $MgCl_2$ and four water molecules. (b) Optimized configuration of $MgCl_2$ and water in free space and (c) optimized configuration inside $C_{70.}$ The dashed circle in (c) refers to the boundary of C_{70}.

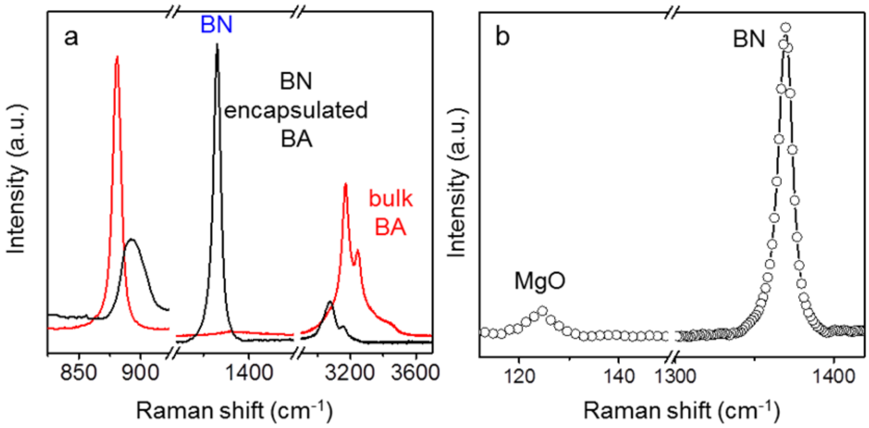

Supplementary Figure 14 | VdW pressure and its effect on BN encapsulation (a) Raman spectrum (514 nm excitation) of bulk BA and BN-encapsulated BA on BN showing the vdW pressure-induced Raman shift. (b) Raman spectrum (488 nm excitation) of BN-encapsulated $MgCl_2$ on BN showing the presence of the MgO band and BN band.

Supplementary Notes

Supplementary Note 1. Atomic force microscopy (AFM) of graphene-encapsulated salt and molecule

Supplementary Fig. 2a shows the AFM image of a GE boric acid (BA) sample on few-layer graphene showing 0.9 nm-thick, large, flat, BA-enclosed regions and small nano-bubbles. Similar to the case of TPA (see the main text), the AFM image shows that the BA-enclosed flat regions are continuous with dimensions reaching up to 10 μm^2 in area. Similar features were also observed in GE-$MgCl_2$ samples. However, unlike images of GE-TPA or BA samples, the AFM image (Supplementary Fig. 2b) taken of a flat, encapsulated region of the GE-$MgCl_2$ showed small bubbles or wrinkle features. We believe that these are caused by gaseous phase by-products produced during the conversion of $MgCl_2$ to MgO (see main text).

We further investigated the possibility of capturing salt and other molecules between the graphene and Si/SiO_2 substrate. Supplementary Fig. 2c shows the AFM image taken of a GE-$MgCl_2$ sample in a region where the $MgCl_2$ is expected to be enclosed between the top single-layer graphene and bottom SiO_2 substrate (indicated by a black square in the inset of Supplementary Fig. 2c). The absence of either bubbles or flat captured regions in this area suggests that the SiO_2/graphene interface does not favour capturing the molecules, possibly due to the relatively high roughness of the Si/SiO_2 substrate; molecules can diffuse along the SiO_2/graphene interface.

Supplementary Note 2. Molecular dynamic simulation of TPA between graphene

The molecular dynamics (MD) simulations were conducted using the GROMACS 4.5.4 package[1]. MD simulations used a list cut-off and short-range coulomb cut-off of 1.3 nm. For the van der Waals (vdW) interactions, we have used a switching function to allow the potential to go to zero. The switching function starts at 0.9 nm and causes the vdW force and potential to go to zero at 1.0 nm. For all simulations, we use a 1 fs time step with the leapfrog integrator[2]. The initial setup for the simulations is shown in Supplementary Fig. 3, where two parallel graphene sheets with a sheet size of 5.16 × 5.10 nm are fixed with an inter sheet separation of 1.3 nm and five TPA molecules are placed within the channel. The remaining volume of the channel was filled with methanol, and the entire channel is placed into contact with a methanol reservoir (8 nm × 8 nm× 8 nm). The graphene sheets are not allowed to bend in the direction perpendicular to the plane of the sheet, which fixes the graphene–TPA/methanol interfacial area. Edges of the channel are open so that the methanol in the reservoir can exchange with the methanol in the channel and reach thermodynamic equilibrium. These simulations were run in the isothermal–isobaric ensemble at a temperature and pressure of 298.15 K and 1 bar, respectively. This was run for 7 ns, with the Berendsen thermostat[3] and barostat[3]. Carbon–hydrogen bond lengths were maintained at 1.08 Å and 1.09 Å for TPA and methanol, respectively, using the LINCS constraint algorithm. The oxygen–hydrogen bond length for methanol was constrained to 0.945 Å using the LINCS[4] constraint algorithm. A Particle–Mesh Ewald (PME)[5,6] summation was used to consider the long-range electrostatics. All of the atomic parameters for the bonding and non-bonding interaction of methanol and TPA were taken

from the OPLS–AA force field[7,8]. The carbon atoms of the graphene sheet had no partial charge but did have Lennard–Jones 12–6 interaction parameters[7,9] of σ_c= 0.355 nm and ε_c= 0.29288 kJ mol^{-1}. This is based on the OPLS–AA parameters for aromatic carbon species. The Lennard–Jones interaction parameters between different atoms were determined using the geometric mixing rules.

After the equilibrium was reached, the reservoirs were removed, and all molecules in the channel were placed into an infinite slit pore in which the graphene sheets were1.3 nm apart, as shown in Supplementary Fig. 3b. The slit pore was seen as infinite due to the periodic boundary conditions applied in the directions parallel to the graphene sheets. The simulation was run in the canonical ensemble with constant box dimensions of 5.1 nm × 5.1 nm × 3.0 nm and at a constant temperature of 298.15 K. To maintain the temperature, a Nosé–Hoover[10,11] thermostat was used with a temperature coupling constant of 0.2 ps. The z-dimension was kept larger than the channel width in order to prevent periodic images from interacting with each other perpendicularly to the graphene sheets. These simulations had an initial energy minimization step to ensure that there were no high-energy overlaps at the start of the simulation. Subsequently, the sheets were pulled together at a rate of $0.0001\, nm\, ps^{-1}$, and the separation was changed from 1.3 nm to lower values. For this simulation, the PME summation is used in only 2 dimensions—those in the plane of the graphene sheet.

The orientations of the TPA molecules with respect to the graphene sheets were measured for different graphene separations as shown in Supplementary Fig. 4. To monitor the change in the orientation of the TPA molecules during the simulations, we have looked at the angle between the vector normal to the graphene sheet and normal to the phenyl rings. This angle can be measured from either of the graphene sheets but has been restricted to always be from 0–90°, allowing the 'TPA angles' to be averaged over all of the molecules. For each point on Supplementary Fig. 4 the TPA angle at a particular inter sheet separation is averaged over 100 snapshots taken at 100 fs time intervals. The error for each point was also calculated, but found to be smaller than the thickness of the line in the plot (Supplementary Fig. 4) so has not been directly included. Around a channel width of 1.1–1.3 nm there is no favourable orientation of the phenyl rings with respect to the plane of the graphene sheets and there is a large variation in the average angle between the phenyl rings and graphene sheets. By compressing the channel width to 0.9–1.0 nm, the phenyl rings are forced to orient themselves perpendicular to the graphene sheet giving higher TPA angles $\approx$ 75° (see Supplementary Fig. 4 inset). Under further compression, at a channel width of 0.7–0.8 nm, the phenyl rings lie at an angle of $\sim$ 45° angle to the graphene sheets, and further confinement leads to alignment of the TPA parallel to graphene sheets (see Supplementary Fig. 4 inset).

From the infinite channel simulations it is possible to determine the pressure on confined molecules between the graphene sheets at different channel widths from the trace of the rank-2 stress sensor[12,13] (see Supplementary Fig. 5). Simulations were further carried out by varying the strength of interaction between the graphene sheets and confined molecules to investigate the effect of interaction strength on the observed vdW pressure inside the nano-enclosures. This was done by changing the Lennard-Jones 12-6

parameters for the C atoms of the graphene sheets. Values of σ_c = 0.321 nm and 0.355 nm were used with values of ε_c = 0.05 kJ mol^{-1}, 0.29 kJ mol^{-1} and 0.60 kJ mol^{-1}, giving 6 combinations of different parameters and this range of values will provide us a better insight on the interactions between confined molecules and graphitic surface. It has been shown that this range of parameters turn graphite surface from hydrophobic to hydrophilic as ε_c varied from 0.05 kJ mol^{-1} to 0.60 kJ mol^{-1} with σ_c = 0.321 nm[14]. The pressure on entrapped liquid molecules was then monitored as a function of the interlayer sheet distance. Since the graphene atoms were kept fixed at their positions, the forces acting on them are not included in the calculation of the pressure. As molecules are compressed into the channel, pressure inside the channel increases (see Supplementary Fig. 5). The plot shows that for interlayer separation below 0.9 nm ca. the pressure starts to increase very rapidly reaching a value of 1 GPa depending on the value of σ_c. Indeed, in a given nano-enclosure the smaller graphene carbon atoms (σ_c = 0.321 nm) reduce the pressure because the confined molecules have more volume to occupy. Increasing the value of ε_c instead induces a modest increase in pressure since the carbon atoms are more attractive at long distances, but more repulsive at shorter distances (i.e. the potential energy increases more rapidly) and therefore at large confinement (below 0.8 nm), where the majority of the interaction with the graphene sheets are repulsive, the pressure increases. Considering that the experimental interlayer distance from the AFM measurements is an average value over an area of few nanometers, and the force field (OLPS) used in the simulation is parameterized to reproduce density of trapped molecules at atmospheric pressure but not at high pressure, our simulation results agree reasonably well with the experimental data.

Supplementary Note 3. Estimation of strain on the top layer graphene

Supplementary Fig. 6 shows the Raman spectra of graphene (collectively from top and bottom layers) obtained from the three different regions of GE-BA sample including the collapsed graphene region, 1-nm-thick flat BA enclosed region and a nanobubble of height 60 nm and base 1.5 μm. G band in the Raman spectrum of collapsed region appears as a single mode due to merging of G band from both the top SLG and bottom FLG, whereas 2D band looks like a convolution because the peak position and intensity of 2D band from SLG and FLG are certainly distinctive. In contrast, Raman spectrum obtained from the 1-nm-thick flat enclosed region and nanobubble shows broadening/splitting of G and 2D bands due to the redshift in the SLG Raman modes caused by the isotropic strain developed in top SLG during the encapsulation. For example, in the case of nanobubble, G peak position is decreased from 1583 cm^{-1} to 1542 cm^{-1} while 2D peak has shifted to 2593 cm^{-1} from 2683 cm^{-1}. Based on the reported Raman shift of G and 2D peak positions with respect to the strain[15], we have estimated the strain developed on top SLG at this bubble and flat 1-nm-thick enclosures as ~ 0.7% and 0.05-0.1% respectively.

Supplementary Note 4. Raman spectrum of $MgCl_2$ upon hydration

$MgCl_2$ is known to be highly hygroscopic, causing the thin $MgCl_2$ films to absorb water molecules quickly from the atmosphere. The Raman spectrum (Supplementary Fig. 7) obtained from a dried thin layer of $MgCl_2$ formed by drop casting the salt solution on Si/SiO_2 and vacuum drying has shown two Raman bands at ≈157 and 240 cm^{-1} which

are characteristic of $MgCl_2$[16]. We have also observed a less intense Raman bands around 3100-3600 cm^{-1} correspond to the O–H bending modes. These modes are expected to arise from the residual water molecules left in the dry $MgCl_2$ thin layer; the very high hygroscopic nature of $MgCl_2$ prevents the water molecules from being evaporated completely. When the dried sample was later exposed to air the above mentioned Raman bands of $MgCl_2$ disappeared, and a new band at ≈ 200 cm^{-1} with a full width half maximum (FWHM) of 60 cm^{-1} appeared along with the high-intensity O–H Raman bands. These observed features are in good agreement with Raman spectra reported[16,17] for the hydrates of $MgCl_2$.

Supplementary Note 5. Defect formation in graphene due to chemical reaction

Supplementary Fig. 8 shows the Raman spectrum of graphene obtained from the GE-$MgCl_2$ sample with the presence and absence of MgO in the enclosure. The appearance of a defect-induced D band at 1345 cm^{-1} and a D′ band at 1620 cm^{-1} from the regions where MgO is present in and absence of these bands in the MgO-absent region indicates that the defects are created in the graphene lattice during the chemical conversion of $MgCl_2$ to MgO. Similar features were also observed in the case of GE-$CuSO_4$ samples. We explain this formation of defects in the graphene as an outcome of aggressive chemical reaction that occurs inside the graphene enclosure. As explained in the main text and above, hydrated $MgCl_2$ trapped inside the graphene enclosure experiences high pressure and undergoes a chemical reaction yielding solid MgO along with the vapours of HCl and H_2O as by-products (while in the case of $CuSO_4$, products are CuO, H_2SO_4 and H_2O). The reactive acids such as HCl and H_2SO_4 are known to create defects in graphene and could explain the observed D-peak[18,19].

It is noteworthy that the observed changes in Raman spectrum and TEM of the GE-salt/molecule samples are not attributed to the intercalation like chemical reactions where certain salts (e.g. $FeCl_3$) and metals (Cs, Ca etc.) form intercalated compounds with graphite and BN[20]. In general, intercalation of salts and metals occurs at high temperatures in the presence of molten salts/metals and it causes the blue shift in peak position of the G-band in graphene Raman spectrum due to increased carrier concentration[20]. Absence of the typical intercalation reaction conditions and absence of the blue shift in G-peak position of graphene rules out the formation of intercalated compounds in our experiments.

Supplementary Note 6. Electron diffraction simulations

Electron diffraction patterns for single, bilayer and trilayer MgO sheets were simulated using the JEMS software[21] using the kinematical approximation (Supplementary Fig. 9a-c). The corresponding atomic models used for these simulations are shown in Supplementary Fig. 9 (d-f). Because of the structure factor of the bulk MgO crystal (rock salt structure, space group $Fm\bar{3}m$, No. 225), the (110) lattice reflection is forbidden. However, these simulations show that for the single-layer case, the forbidden (110) reflection is present, whereas in the bilayer and trilayer cases, this reflection is absent and extremely weak, respectively. Our simulation also shows that the (110) reflections will be present for MgO crystals with an odd number of layers, although the intensity decreases rapidly with increasing sheet thickness.

Supplementary Fig. 10a and 10b shows a HRTEM image obtained from the thinnest MgO crystals encapsulated between two graphene sheets and the corresponding fast Fourier transform (FFT) image showing the lattice spacing corresponding to the graphene sheets and to the MgO crystals. The presence of (110) MgO reflections suggests that the MgO crystals are single layers. We have not clearly observed the (110) forbidden reflections in the experimental diffraction pattern shown in Fig. 3 in the main text. This may be due to the weak intensity of the (110) reflection, which is approximately 10 times lower than the (200) reflections for monolayer materials (see Supplementary Fig. 9a). Additionally, the experimental diffraction patterns are acquired over a much larger area (~ 1 μm^2), where the thickness is non-uniform and which predominantly contains few layers of MgO. Consequently, the diffraction signal from the monolayer MgO is expected to be low and is likely to be below the signal-to-background of our experimental diffraction conditions. The forbidden reflections appear in FFT taken from HRTEM images as here the measured interference pattern comes from a highly localized area, which contains a much higher proportion of monolayer material, allowing the weak (110) reflections to be visible in the power spectrum of the HRTEM image.

Supplementary Note 7. Other graphene-encapsulated salts and compounds ($Ca(OH)_2$, CsI and $CuSO_4$)

We have also performed encapsulation experiments using aqueous solutions of $Ca(OH)_2$, CsI and $CuSO_4$ and investigated the resulting materials using Raman spectroscopy and STEM analysis. These experiments provided further insight into the mechanism of the vdW pressure induced chemical changes observed above. Supplementary Fig. 11a compares the Raman spectrum of GE-$Ca(OH)_2$, to bulk $Ca(OH)_2$ and CaO prepared by annealing $Ca(OH)_2$ at 350 °C in vacuum. The spectrum obtained from bulk $Ca(OH)_2$ shows Raman modes at 357, 678, 1087 and 3620 cm^{-1} all of which are assigned to pure $Ca(OH)_2$[22] except the peak 1087 cm^{-1}, which appears due to the small amount of inevitable $CaCO_3$ traces[22]. The Raman spectrum obtained from flat regions of the GE-$Ca(OH)_2$ sample show different features compared with bulk $Ca(OH)_2$. Interestingly, it shows similar signatures of the Raman spectrum of CaO with small traces of $Ca(OH)_2$[22]. Apart from the weak traces of $Ca(OH)_2$, the bands at 1090, 1785 and 1933 cm^{-1} in the encapsulated samples are due to the luminescence bands of CaO[22]. All of these observed Raman features in the encapsulated samples are in good agreement with the reference CaO sample as shown in Supplementary Fig. 11a.

Our TEM analysis of GE-$Ca(OH)_2$ samples further confirms the transformation of $Ca(OH)_2$ to CaO. Supplementary Fig. 11b shows the HRTEM image of atomically thin CaO crystals formed in the flat GE regions of the $Ca(OH)_2$ samples. The observed lattice constant of CaO (≈ 2.4 Å) is consistent with the reported lattice constant of bulk CaO. Similar to the case of MgO, the forbidden (110) lattice reflections (marked with red circles) are visible in the FFT of thin CaO crystals (inset of Supplementary Fig. 11b). This implies that the thinnest CaO crystals found in the flat encapsulated regions are monolayers. All the above observations confirm that the decomposition of hydroxide compounds into oxides is possible at room temperature inside nanometer thick graphene enclosures due to the presence of GPa vdW pressure. Similar room-temperature

decomposition of hydroxide compounds were reported before in mechanochemistry, where hydroxide compounds were decomposed by grinding with SiO_2[23], demonstrating that mechanical force can provide the energy needed to initiate the decomposition as an alternative to conventional thermal energy.

We have also studied GE-CsI and $CuSO_4$ samples using TEM and Raman spectroscopy. Supplementary Fig. 12a shows the high angle annular dark field (HAADF) STEM image and EELS analysis of a GE-CsI sample. Unlike $MgCl_2$, these measurements are consistent with bulk CsI and we have not found any evidence for chemical reaction in the case of this salt. This can be explained by the absence of a hydrolysis reaction in CsI, as observed in the case of $MgCl_2$. When CsI is dissolved in water, the relative proportions of H^+ and OH^- ions in solution remain unchanged, and the solution remains neutral. In other words, the salts of strong acids and bases are completely ionized in water and do not undergo hydrolysis[24]. $CuSO_4$ is an example of a salt of a weak base and a strong acid and this compound undergoes hydrolysis in a similar way to $MgCl_2$. Our encapsulation experiments for $CuSO_4$ indicate that here again chemical changes result due to vdW pressure between the graphene sheets. Supplementary Fig. 12b shows the Raman spectrum obtained from bulk $CuSO_4$ (pentahydrate) and GE-$CuSO_4$. The spectrum of bulk $CuSO_4$ shows the major Raman bands corresponding to symmetric stretching (ν_1) vibration at 984 cm^{-1}, the symmetric bending (ν_2) modes between 425–468 cm^{-1}, the antisymmetric stretching (ν_3) modes at 1097 and 1143 cm^{-1}, the antisymmetric bending (ν_4) mode at the 613 cm^{-1} and 248–283 cm^{-1} bands due to the internal modes of the complex. In addition, we have also observed broad bands at 3200–3500 cm^{-1} due to water stretching modes. All of these observed Raman bands are in good agreement with previously reported studies[25,26]. However, the Raman spectra from flat, 1-nm-thick regions of GE-$CuSO_4$ show different Raman bands at 301, 346 and 627 cm^{-1}, which are in good agreement with the Raman modes of copper oxide (CuO), namely, 297 cm^{-1} A_g mode, 344 cm^{-1} B_{1g} mode and 629 cm^{-1} B_{2g} mode[27].

Supplementary Note 8. Stability of nano-enclosed salts

To investigate the stability of enclosed hydrated $MgCl_2$, we performed density functional theory (DFT) calculations using the GAUSSIAN package (G09)[28]. This is an electronic-structure package that uses Gaussian type orbitals as a basis; in this study, the 6-31G* basis set was used. The exchange-correlation (XC) hybrid functional M06-2X was used in G09. The combination of the basis set and XC functional are expected to produce a reliable description of the electronic properties of our system. The self-consistency loop iterates over small changes in the atomic positions until the change in the total energy is less than 10^{-7} eV. The geometries are then considered to be relaxed once the force on each nucleus is less than 50 meV/Å. Natural bonding orbital (NBO) analysis[29] was performed with the M06-2X/6-31G* level of theory.

Here, we have used a C_{70} fullerene molecule as a hydrophobic confinement system. This is a simple model for studying trapped molecules inside a hydrophobic cage, which is computationally less expensive than a graphene enclosure. The initial configuration of the $MgCl_2$ and water molecules in free space is shown in Supplementary Fig. 13(a), where four water molecules are randomly placed around two $MgCl_2$. The final optimized

configurations in free space and within the confinement are shown in Supplementary Fig. 13(b) and (c), respectively. In free space, the starting structure for H_2O and $MgCl_2$ was compact, as shown in Supplementary Fig. 13a. At the end of the structure optimization, the increased separation between H_2O and $MgCl_2$, as shown in Supplementary Fig. 13b, suggests that the final free space structure is still four H_2O molecules and two $MgCl_2$. Within the confinement, C_{70}, H_2O and $MgCl_2$ are all included in the optimization, and the final configuration includes MgO, 2HCl, $2H^+$ and $2Cl^-$ ions, as shown in Supplementary Fig. 13c. Each O atom in the Mg–O bonds should be saturated by a H atom. These additional –H bonds guarantee the convergence of the DFT calculations because the valance of the oxygen atom is 2. These H atoms would be replaced by other Mg atoms if a larger volume with more Mg atoms was simulated. The Mg–O bond lengths in our DFT optimization are in the range 1.88–1.946 Å, which agrees with the bulk MgO lattice constant (≈ 2 Å). We do not claim that this optimization procedure is equivalent to the precise reaction mechanism; however, it explicitly shows that salts enclosed in extremely small volumes are unstable and behave differently from their bulk counterparts**.** We have also performed additional DFT calculations using confined $Mg(OH)_2$ and three water molecules inside a C_{70}. The optimized configuration consists of four Mg–O bonds (terminated with H), one H_2O and one H_2. These simulations further show that the MgO is energetically more stable inside extremely small volumes than $MgCl_2$ and $Mg(OH)_2$. Similar structural changes were also observed when we used C_{90} or C_{180} as the molecular enclosure system.

To study the effects of confinement on CsI, we performed similar DFT calculations for two CsI and six water molecules enclosed in C_{90}. After optimization, we do not see any signature of Cs–O bond formation; instead, all the oxygen atoms of water prefer to be far from the Cs ion, and the optimized structure includes $2Cs^+$, $2I^-$ ions together with $2OH^-$, $2H^+$ and four water molecules. This further agrees with our experimental observations and supports the proposed reaction mechanism.

Supplementary Note 9. Proposed $MgCl_2$ Reaction Mechanism

Magnesium chloride is one of the typical ionic halides that has various hydrated states $MgCl_2(H_2O)_x$. Magnesium chloride hexahydrate, $MgCl_2(H_2O)_6$, is a common hydrate, and it can be simultaneously dehydrated and decomposed at 415 °C to produce MgO[30]. Magnesium salts can also be converted to $Mg(OH)_2$ through a hydrothermal reaction and a subsequent calcination produces MgO[31,32]. Because $MgCl_2(H_2O)_6$ is soluble in water, the corresponding equilibrium between the solid and liquid phases is as follows:

$$MgCl_2(6H_2O) \rightleftarrows Mg^{2+}(aq) + 2Cl^-(aq) + 6H_2O \qquad (1)$$

The ionization of water strongly increases with increasing pressure. K_w (the ionic product or ionization constant of water) increases by two orders of magnitude when the pressure increases from 0.1 MPa to 1 GPa[33]. Therefore, we have an additional reaction in water when the pressure is high, expressed as

$$H_2O \rightarrow H^+ + OH^- \qquad (2)$$

Because $MgCl_2$ is a salt composed of a weak base and a strong acid, when these salts are dissolved in water, they produce acidic solution and undergo hydrolysis[24]. Mg^{2+} ions in the aqueous solution react with the available OH^- ions from the ionization of water to form $Mg(OH)_2$. Consequently, the mechanism that is assumed to lead to the formation of MgO precipitates in our experiment in the presence of high vdW pressure (T = 298K, P ≈ 1.2 GPa) can be separated into two steps:

i) $Mg^{2+}+2(OH^-) \rightarrow Mg(OH)_2$ (3)

$Cl^- + H^+ \rightarrow HCl$ (4)

ii) $Mg(OH)_2 \rightarrow MgO + H_2O$ (5)

The first step results in the production of a salt hydroxide and HCl, which is due to the salt hydrolysis reaction. The second reaction is the decomposition of hydroxide to oxide and is due to the confinement and pressure effects caused by the encapsulation (similar to the $Ca(OH)_2$ case shown above). The two steps can be combined into a single reaction mechanism:

$$MgCl_2(6H_2O) \rightarrow MgO+5H_2O+2HCl \quad (6)$$

Gibbs free energy calculations using the standard free energy of formation explain that the above proposed reaction is not feasible at ambient conditions (room temperature and ordinary atmospheric pressure (0.1 MPa)) in terms of reaction energy. This indicates the importance of confinement and pressure in our experiments for lowering the activation barrier for the reaction to occur at room temperature. Dedicated DFT calculations are required for the quantitative analysis of reaction energy in order to understand the exact reaction mechanism.

Supplementary Note 10. VdW pressure and its effects on other 2D crystal enclosures

To examine the presence of vdW pressure and its effects in other graphene-like 2D crystals, Raman experiments have also been performed on thin BN-encapsulated molecules/salts on few-layer BN. Supplementary Fig. 14 shows the Raman spectrum of BN-encapsulated BA on few-layer BN. A clear blue shift of 8.8–10 cm^{-1} in the ν_3 (A_g) symmetric B–O bond stretching mode and an apparent red shift in the O–H bond stretching modes are observed for ≈ 1-nm-thick flat regions of BN-encapsulated BA. From the observed shift in ν_3 (A_g) mode, the estimated vdW pressure in BN nano-enclosures is ≈ 1.9–2.2 GPa, which is slightly higher than that of the graphene nano-enclosures. This increased pressure could be attributed to the strong vdW interactions between BN sheets along with additional charge–charge interactions due to the polar nature of BN. In corroboration with the above experiment, the Raman spectrum of BN-encapsulated $MgCl_2$ has also shown the Raman band at 125 cm^{-1} assigned to MgO, similar to the case of GE-$MgCl_2$ samples.

As an additional example, we have studied thin mica-encapsulated BA and $MgCl_2$ on mica samples; however, we have not found any signatures of the trapped molecules or salts between the mica sheets. The only signatures we found in our Raman study was the presence of water (O–H band at 3100–3600 cm^{-1}) between the mica layers. We attribute

this to the high hydrophilicity of mica, which favors the formation of adlayers of water[34,35] and hinders other molecules or salts from being trapped at the interface.

Supplementary References

1 Van der Spoel, E. L. D. *et al. Gromacs user manual,* version 4.5 (2011).

2 Hockney, R. W., Goel, S. P. & Eastwood, J. W. Quiet high-resolution computer models of a plasma. *J. Comput. Phys.* **14**, 148-158 (1974).

3 Berendsen, H. J. C., Postma, J. P. M., Gunsteren, W. F. v., DiNola, A. & Haak, J. R. Molecular dynamics with coupling to an external bath. *J. Chem. Phys.* **81**, 3684-3690 (1984).

4 Hess, B., Bekker, H., Berendsen, H. J. C. & Fraaije, J. G. E. M. LINCS: A linear constraint solver for molecular simulations. *J. Comput. Chem.* **18**, 1463-1472 (1997).

5 Ulrich, E. *et al.* A smooth particle mesh Ewald method. *J. Chem. Phys.* **103**, 8577-8593 (1995).

6 Darden, T., York, D. & Pedersen, L. Particle mesh Ewald: An N·log(N) method for Ewald sums in large systems. *J. Chem. Phys.* **98**, 10089-10092 (1993).

7 Jorgensen, W. L., Maxwell, D. S. & Tirado-Rives, J. Development and testing of the OPLS all-atom force field on conformational energetics and properties of organic liquids. *J. Am. Chem. Soc.* **118**, 11225-11236 (1996).

8 Rizzo, R. C. & Jorgensen, W. L. OPLS all-atom model for amines: Resolution of the amine hydration problem. *J. Am. Chem. Soc.* **121**, 4827-4836 (1999).

9 Joshi, R. K. *et al.* Precise and ultrafast molecular sieving through graphene oxide membranes. *Science* **343**, 752-754 (2014).

10 Nosé, S. A molecular dynamics method for simulations in the canonical ensemble. *Mol. Phy.* **52**, 255-268 (1984).

11 Hoover, W. G. Canonical dynamics: Equilibrium phase-space distributions. *Phys. Rev. A* **31**, 1695-1697 (1985).

12 Irving, J. H. & Kirkwood, J. G. The statistical mechanical theory of transport processes .IV. The equations of hydrodynamics. *J. Chem. Phys.* **18**, 817-829 (1950).

13 Todd, B. D., Evans, D. J. & Daivis, P. J. Pressure tensor for inhomogeneous fluids. *Phys. Rev. E* **52**, 1627-1638 (1995).

14 Werder, T., Walther, J. H., Jaffe, R. L., Halicioglu, T. & Koumoutsakos, P. On the water-carbon interaction for use in molecular dynamics simulations of graphite and carbon nanotubes. *J. Phys. Chem. B* **107**, 1345-1352 (2003).

15 Zabel, J. *et al.* Raman spectroscopy of graphene and bilayer under biaxial strain: Bubbles and balloons. *Nano Lett.* **12**, 617-621 (2012).

16 Capwell, R. J. Raman spectra of crystalline and molten $MgCl_2$. *Chem. Phys. Lett.* **12**, 443-446 (1972).

17 Voigt, W. K., Peter. Raman spectroscopic evidence for cation-anion contact in molten hydrates of magnesium chloride. *Acta Chem. Scand.* **A40**, 354-357 (1986).

18 Coleman, V. A. *et al.* Defect formation in graphene nanosheets by acid treatment: an x-ray absorption spectroscopy and density functional theory study. *J. Phys. D: Appl. Phys.* **41,**062001 (2008).

19 Wang, Y. Y. *et al.* Structural evolution in CVD graphene chemically oxidized by sulphuric acid. *J. Raman Spectrosc.* **46**, 283-286 (2015).

20 Dresselhaus, M. S. & Dresselhaus, G. Intercalation compounds of graphite. *Adv. Phys.* 51, 1–186 (2002).

21 Stadelmann, P. A. EMS - a software package for electron diffraction analysis and HREM image simulation in materials science. *Ultramicroscopy* **21**, 131-145 (1987).

22 Schmid, T. & Dariz, P. Shedding light onto the spectra of lime: Raman and luminescence bands of CaO, $Ca(OH)_2$ and $CaCO_3$. *J. Raman Spectrosc.* **46**, 141-146 (2015).
23 Liao, J. & Senna, M. Mechanochemical dehydration and amorphization of hydroxides of Ca, Mg and Al on grinding with and without SiO_2. *Solid State Ionics* **66**, 313-319 (1993).
24 Malone, L. J. & Dolter, T. O. *Basic Concepts of Chemistry* (Wiley, Newyork, ed. 9, 2012).
25 Fu, X., Yang, G., Sun, J. & Zhou, J. Vibrational spectra of copper sulfate hydrates investigated with low-temperature Raman spectroscopy and terahertz time domain spectroscopy. *J. Phys. Chem. A* **116**, 7314-7318 (2012).
26 Liu, D. & Ullman, F. G. Raman spectrum of $CuSO_4 \cdot 5H_2O$ single crystal. *J. Raman Spectrosc.* **22**, 525-528 (1991).
27 Hagemann, H., Bill, H., sadowski, W., Walker, E. & François, M. Raman spectra of single crystal CuO. *Solid State Commun.* **73**, 447-451 (1990).
28 M. J. Frisch et al. *GAUSSIAN 1998, http://www.gaussian.com/.*
29 Reed, A. E., Curtiss, L. A. & Weinhold, F. Intermolecular interactions from a natural bond orbital, donor-acceptor viewpoint. *Chem. Rev.* **88**, 899-926 (1988).
30 Huang, Q., Lu, G., Wang, J. & Yu, J. Thermal decomposition mechanisms of $MgCl_2 \cdot 6H_2O$ and $MgCl_2 \cdot H_2O$. *J. Anal. Appl. Pyrolysis* **91**, 159-164 (2011).
31 Ding, Y. *et al.* Nanoscale magnesium hydroxide and magnesium oxide powders: Control over size, shape, and structure via hydrothermal synthesis. *Chem. Mater.* **13**, 435-440 (2001).
32 McKelvy, M. J., Sharma, R., Chizmeshya, A. V. G., Carpenter, R. W. & Streib, K. Magnesium hydroxide dehydroxylation: In situ nanoscale observations of lamellar nucleation and growth. *Chem. Mater.* **13**, 921-926 (2001).
33 Marshall, W. L. & Franck, E. U. Ion product of water substance, 0–1000 °C, 1–10,000 bars new international formulation and its background. *J. Phys. Chem. Ref. Data* **10,** 295-304 (1981).
34 Xu, K., Cao, P. & Heath, J. R. Graphene visualizes the first water adlayers on mica at ambient conditions. *Science* **329**, 1188-1191 (2010).
35 Severin, N., Lange, P., Sokolov, I. M. & Rabe, J. P. Reversible dewetting of a molecularly thin fluid water film in a soft graphene–mica slit pore. *Nano Lett.* **12**, 774-779 (2012).
